# Monkeypox virus A29L protein as the target for specific diagnosis and serological analysis

**DOI:** 10.1007/s00253-024-13361-6

**Published:** 2024-11-21

**Authors:** Chia-Yu Liang, Tai-Ling Chao, Chong‐Syun Chao, Wang-Da Liu, Yu-Chen Cheng, Sui-Yuan Chang, Shih-Chung Chang

**Affiliations:** 1 Department of Biochemical Science and Technology, College of Life Science, National Taiwan University, Taipei, 106 Taiwan; 2 Department of Clinical Laboratory Sciences and Medical Biotechnology, College of Medicine, National Taiwan University, Taipei, 100 Taiwan; 3 Department of Internal Medicine, College of Medicine, National Taiwan University Hospital, National Taiwan University, Taipei, 100 Taiwan; 4 Department of Medicine, National Taiwan University Cancer Center, Taipei 106, Taiwan; 5 Department of Laboratory Medicine, College of Medicine, National Taiwan University Hospital, National Taiwan University, Taipei, 100 Taiwan; 6 Center of Biotechnology, National Taiwan University, Taipei, 106 Taiwan

**Keywords:** Monkeypox (Mpox), Monkeypox virus (MPXV), A29L protein, Monoclonal antibody, Lateral flow immunochromatographic assay, Serological assay

## Abstract

**Abstract:**

The unexpected monkeypox (Mpox) outbreak has been reported in many non-endemic countries and regions since May 2022. The mutant strains of Mpox virus (MPXV) were found with higher infectivity and greater capability for sustained human-to-human transmission, posing a significant public health threat. MPXV A29L, a protein homolog of vaccinia virus (VACV) A27L, plays an important role in viral attachment to host cell membranes. Therefore, MPXV A29L is considered the diagnostic target and the potential vaccine candidate for eliciting neutralizing antibodies and protective immune responses. In response to the escalating Mpox outbreak, three monoclonal antibodies (mAbs) (2-9B, 3-8G, and 2-5H) targeting the different domains of MPXV A29L have been developed in the study. Among them, 2-5H is highly specific for MPXV A29L without exhibiting cross-reactivity with VACV A27L. The antibody pairing composed of 2-5H and 3-8G has been developed as the lateral flow immunochromatographic assay for specific detection of MPXV A29L. However, these three mAbs were unable to inhibit A29L binding to heparin column or prevent MPXV infection in the neutralization test assays. The results of the serological assays using the truncated A29L fragments as the antigens showed that the Mpox patient sera contained significantly lower levels of antibodies targeting the N-terminal 1–34 residues of A29L, suggesting that the N-terminal portion of A29L is less immunogenic upon natural infection.

**Key points:**

*• MAbs 2-9B, 3-8G, and 2-5H neither interrupted A29L binding to heparin nor neutralized MPXV.*

*• The LFIA composed of 3-8G and 2-5H can specifically distinguish MPXV A29L from VACV A27L.*

*• Mpox patient sera contained lower levels of antibodies targeting the N-terminal portion of A29L.*

## Introduction

Monkeypox (Mpox) is a zoonotic infectious disease traditionally confined to Central and West Africa. However, the unexpected global Mpox outbreak in 2022 has swiftly disseminated to numerous non-endemic countries and regions where the mutant strains were found to exhibit increased infectivity and enhanced capability for sustained human-to-human transmission (Ferdous et al. 2023; Li et al. 2022b; Okwor et al. 2023). The causative agent of the Mpox disease is Mpox virus (MPXV), an enveloped double-stranded DNA virus belonging to the *Orthopoxvirus* genus of the Poxviridae family. MPXV comprises two distinct lineages: the Western African clade and the Central African clade (Papukashvili et al. 2022; Petersen et al. 2019). The prevailing mutant strain for the 2022 Mpox epidemic originated from the West African branch, which was less severe and lethal relative to the Central African branch, and mostly caused mild clinical symptoms including mild rash, fever, pruritus, myalgia, headache, skin ulcer, abdominal symptom, pharyngitis, nausea or vomiting, and conjunctivitis (Cho et al. 2024). Nevertheless, it exhibited a higher mutation rate than anticipated (Isidro et al. 2022), potentially leading to increased transmissibility and virulence. Due to the protein-coding genes being highly conserved among members of the *Orthopoxvirus* genus (Shchelkunov et al. 2001), previous studies have indicated that the smallpox vaccine effectively prevented infections caused by other orthopoxviruses in animals, including MPXV (Hatch et al. 2013; Hooper et al. 2004). Both early and recent studies have reported that individuals vaccinated with smallpox vaccine exhibited 85–87% protection against MPXV infection (Christodoulidou and Mabbott 2023; Di Giulio and Eckburg 2004). However, the efficacy of the smallpox-based vaccine against the MPXV mutant strain could potentially diminish over time as the virus evolves to evade human immunity (Huang et al. 2022; Wang et al. 2023a). Moreover, the immune durability and protective effects of the smallpox vaccine might have waned in the elderly and unvaccinated young populations. Importantly, severe cases of Mpox can still lead to fatalities, posing an imminent threat to public health. Hence, there is an urgent need to develop specific detection tools, novel vaccines, and therapeutic drugs to effectively monitor and control the emerging Mpox outbreak.

The process of MPXV infection consists of four stages, which are conserved across all poxviruses (Senkevich et al. 2005): viral particle entry, fusion, replication, and release (Wang et al. 2023b). Like other poxviruses, MPXV possesses two distinct infectious virion forms: intracellular mature virus (IMV) and extracellular enveloped virus (EEV). Both earlier and recent investigations have identified M1R, H3L, E8L, and A29L proteins of MPXV as crucial IMV antigens responsible for eliciting neutralizing antibodies (Gilchuk et al. 2016; Moss 2011; Yefet et al. 2023; Zajonc 2017). These antibodies play a pivotal role in influencing the fusogenic properties of IMVs, interrupting viral adhesion to the host cell surfaces, and impeding viral entry (Benhnia et al. 2009a, 2009b; Hubert et al. 2023; Lustig et al. 2004; Putz et al. 2006). The A29L protein of MPXV, akin to vaccinia virus (VACV) A27L, plays a crucial role in viral attachment to the host cell membrane, which was facilitated by strong binding affinity of its heparan-binding domain (HBD) with heparan sulfate and heparan sulfate-derived oligosaccharides (Hsiao et al. 1998; Shi et al. 2022). Consequently, both VACV A27L and MPXV A29L have been identified as potential vaccine candidates for elucidating neutralizing antibodies and protecting hosts from viral challenges in animal models (Berhanu et al. 2008; Heraud et al. 2006; Li et al. 2023; Riccardo and Pablo 2023; Sang et al. 2023; Tang et al. 2023; Xia et al. 2023; Zhang et al. 2023). In addition, they serve as target antigens for serological assays and specific detection of orthopoxvirus infections (Cohn et al. 2023; Stern et al. 2016; Yang et al. 2023).

In the study, the recombinant MPXV A29L protein with heparin-binding ability was purified from *Escherichia coli* (*E. coli*) expression system and used to immunize BALB/c mice for generating monoclonal antibodies (mAbs). Subsequently, three anti-A29L mAbs, 2-9B, 3-8G, and 2-5H, were produced and further subjected to determine their cross-reactivity with VACV A27L and to access their ability of neutralizing MPXV infection. The antibody pairing, specifically 2-5H and 3-8G, was utilized in the lateral flow immunochromatographic assay (LFIA) for specific detection of MPXV A29L. Additionally, the truncated fragments of A29L were generated for epitope mapping experiments and serological assays to evaluate the antibody profile of the serum samples from Mpox patients.

## Materials and methods

### Preparation of the recombinant MPXV A29L and VACV A27L

The cDNA encoding for MPXV A29L (GenBank: AAQ72865.1) or VACV A27L (Swiss-Prot: P11258.3) was synthesized (Synbio Technologies, USA) and then subcloned into the pET28a vector (Novagen) with a C-terminal hexa-histidine tag (His-tag). The pET28a-A29L-His or pET28a-A27L-His vector was transformed into *E. coli* BL21(DE3) competent cells and cultured in Luria–Bertani medium containing kanamycin (50 µg/mL). Protein expression was induced by adding isopropyl-1-thio-beta-d-galactopyranoside to the culture medium at a final concentration of 1 mM. After 16 h of induction at 18°C, bacterial cells were collected by centrifugation at 6000 × *g* for 30 min. The cell pellet was resuspended with buffer A (10 mM sodium phosphate, 20 mM imidazole, 0.5 M NaCl, pH 7.4) and homogenized by a cell disruptor (Constant Systems Limited, UK). The His-tagged A29L or A27L was purified by using the HisTrap FF column (Cytiva) with a 10–500 mM gradient of imidazole in buffer A. The purified His-tagged A29L or A27L was stored in phosphate-buffered saline (PBS) and the protein concentration was determined by the Bradford protein assay (Bio-Rad).

### Preparation of the truncated A29L fragments

The cDNA encoding for the truncated A29L fragment (2–20, 2–34, 2–41, 2–84, or 2–110) was subcloned into the pET-28a vector with an N-terminal His-tag and a superfolder green fluorescent protein (sfGFP) tag (Pedelacq et al. 2006). The protein expression and purification procedures were performed as described previously.

### Enzyme-linked immunosorbent assay

The 96-well plate was coated with 100 ng of the purified MPXV A29L in each well and blocked with 0.25% gelatin in PBS buffer containing 0.05% Tween-20 (PBST). The mouse antiserum, cell culture medium, or the purified antibody sample was added to the well and incubated at 37°C for 1 h. After washing three times with PBST, the horseradish peroxidase (HRP)-conjugated goat anti-mouse IgG (H + L) secondary antibody (KPL, USA) was added to the well and incubated at room temperature for an additional 1 h. After washing three times with PBST, 50 μL of 3,3′,5,5′-tetramethylbenzidine substrate (BD Bioscience, USA) was utilized for signal detection, and 50 μL of 2 N H_2_SO_4_ was added to terminate the reactions. The absorbance at 450 nm was measured by Multiskan FC Microplate Photometer (Thermo Fisher Scientific, USA) to record the results. The antibody concentration for reaching the half-maximum absorbance at 450 nm was designated as the *K*_D_ value.

### Immunization of antigen and generation of mouse mAb

The animals’ care and protocol have been reviewed and approved by the Institutional Animal Care and Use Committee (IACUC) of National Taiwan University (Approval Number: NTU-112-EL-00080). The procedures for immunization of antigen and generation of hybridomas were performed as described previously with slight modifications (Cheng and Chang 2021; Lai et al. 2022; Li et al. 2022a). In brief, BALB/c male mice were immunized three times through intraperitoneal injection with 75 μg of purified recombinant MPXV A29L supplemented with Freund’s adjuvant (Sigma-Aldrich, USA) in a 2-week interval followed by a final booster injection of 75 μg purified recombinant MPXV A29L in PBS. The cell fusion step was performed by mixing Sp2/0-Ag14 cells (ATCC CRL-1581) with mouse splenocytes in the presence of polyethylene glycol 1500 (PEG 1500) (Sigma-Aldrich, USA) and then incubated for 2 min with gentle shaking in a 37°C water bath. The additional 10 mL of Dulbecco’s modified Eagle medium (DMEM) was then added to quench the cell fusion step. Cells were collected by centrifugation and resuspended with 30 mL of DMEM containing 15% fetal bovine serum, 1% penicillin–streptomycin, 1 mM sodium pyruvate (Thermo Fisher Scientific, USA), and HAT Media Supplement (Sigma-Aldrich, USA). Cell samples (0.1 mL) were aliquoted into 96-well cell culture plates and grown at 37°C in a 5% CO_2_ incubator. On day 7 post cell fusion, HT Media Supplement (50 μL) was added to each well. The culture media were collected on day 14 for analysis of the target antibodies by enzyme-linked immunosorbent assay (ELISA) using 100 ng of the purified recombinant A29L protein as the antigen. The positive hybridoma cells were further subcloned into mAbs by limiting dilution method.

### Purification of mouse mAb

The hybridoma cell culture media were filtered with a 0.45-µm membrane disc and then loaded on a HiTrap Protein G HP column (Cytiva) which was pre-equilibrated with 20 mM sodium phosphate (pH 7.0). Antibody samples were eluted with 0.1 M glycine–HCl (pH 2.7) and immediately neutralized with 1 M Tris–HCl (pH 9.0). The purified antibody samples were loaded on the PD-10 desalting column (Cytiva) for exchanging buffer with PBS and then stored at − 20 °C before use.

### Heparin column chromatography

The purified MPXV A29L (170 µg) was incubated with the purified mAb (500 µg) overnight at 4°C. The mixture was then loaded on a HiTrap Heparin HP column (Cytiva) which was pre-equilibrated with binding buffer (10 mM sodium phosphate, pH 7.0). After washing the column, 1.5 M NaCl in the binding buffer was applied to elute the bound fractions. The collected fractions were analyzed by SDS-PAGE under the reduction condition, followed by coomassie staining to visualize A29L, antibody heavy chain, and antibody light chain.

### Neutralization test assay

The neutralization test (NT) assay was performed in 96-well tissue culture plates. One day prior to viral infection, Vero E6 cells (ATCC CRL-1586) were seeded at 5 × 10^4^ cells/well in DMEM supplemented with 10% FBS, 100 units/mL penicillin G sodium, 100 μg/mL streptomycin sulfate, and 250 ng/mL amphotericin B (Gibco, USA) at 37°C in the 5% CO_2_ incubator. The MPXV virions (100 TCID_50_) were incubated with antibodies (200 µg/mL or 100 µg/mL) for 2 h at 37°C. The virus-antibody mixtures were then added to the Vero E6 cell monolayers cultured at 37°C in the 5% CO_2_ incubator. After 7 days, cells were fixed with 10% formaldehyde overnight. After removing the media, cells were stained with 0.5% crystal violet and the NT_50_ were calculated.

### Plaque reduction neutralization test

Approximately 2 × 10^5^ Vero E6 cells per well were seeded in 24-well plates in DMEM supplemented with 10% FBS and antibiotics 1 day before infection as described previously. The antibody sample was incubated with 50 PFU of MPXV in the absence or presence of 10% baby rabbit complements (31,061–1, Pel-Freez, USA) at 37°C for 1 h. The mixture was then added to the Vero E6 cell monolayers and incubated for 1 h. After incubation, the mixture was removed and an overlay medium was added, followed by 7 days of incubation. The cell monolayer was then fixed with 10% formalin (equivalent to 3.7% formaldehyde) overnight. After the removal of formalin, the cells were stained with 0.5% crystal violet.

#### LFIA

The anti-MPXV A29L mAb 2-9B or 2-5H was mixed with 40 nm colloidal gold (DCN, O.D.520 = 1) in borate buffer (pH 9.0) at room temperature for 30 min and then blocked with 10% BSA. The mAb-conjugated colloidal gold was resuspended in 20 mM Tris–HCl (pH 8.2) containing 0.05% PEG 20,000, 10% sucrose, and 0.05% sodium azide. A conjugate pad (Cytiva) was pretreated with 20 mM Tris–HCl (pH 8.2) containing 1% BSA, 6% trehalose, 4% sucrose, and 0.05% sodium azide, and then coated with the mAb-conjugated colloidal gold. The capture mAb 3-8G was coated in the test line of the nitrocellulose (NC) membrane (Sartorius UniSart CN140 backed membrane). The protein G was coated in the control line of the NC membrane, which was then blocked with 0.5% casein in 50 mM borate buffer (pH 8.5) at room temperature for 30 min. The NC membrane was subsequently washed with 0.5% sucrose in 50 mM Tris–HCl (pH 7.5) and air-dried at 50 °C for 30 min. The NC membrane was assembled with a sample pad (Cytiva), a adsorbent pad (CF5, Cytiva), and a type-T rapid test cassette. The LFIA kit was stored in the electric autodryer before use.

### Human sera

Human sera were collected from the Mpox patients on day 14 since they were admitted to the hospital and diagnosed with MPXV infection by real-time quantitative polymerase chain reaction. The Mpox patients included in the study have not ever received the smallpox-based vaccine. The collected sera were heated at 56°C for 30 min, and then stored at 4°C. A written informed consent was obtained from all individual patients in the study, which was approved by the Institutional Review Board of National Taiwan University Hospital.

## Results

### Immunization of mice with MPXV A29L protein

**Fig. 1:**
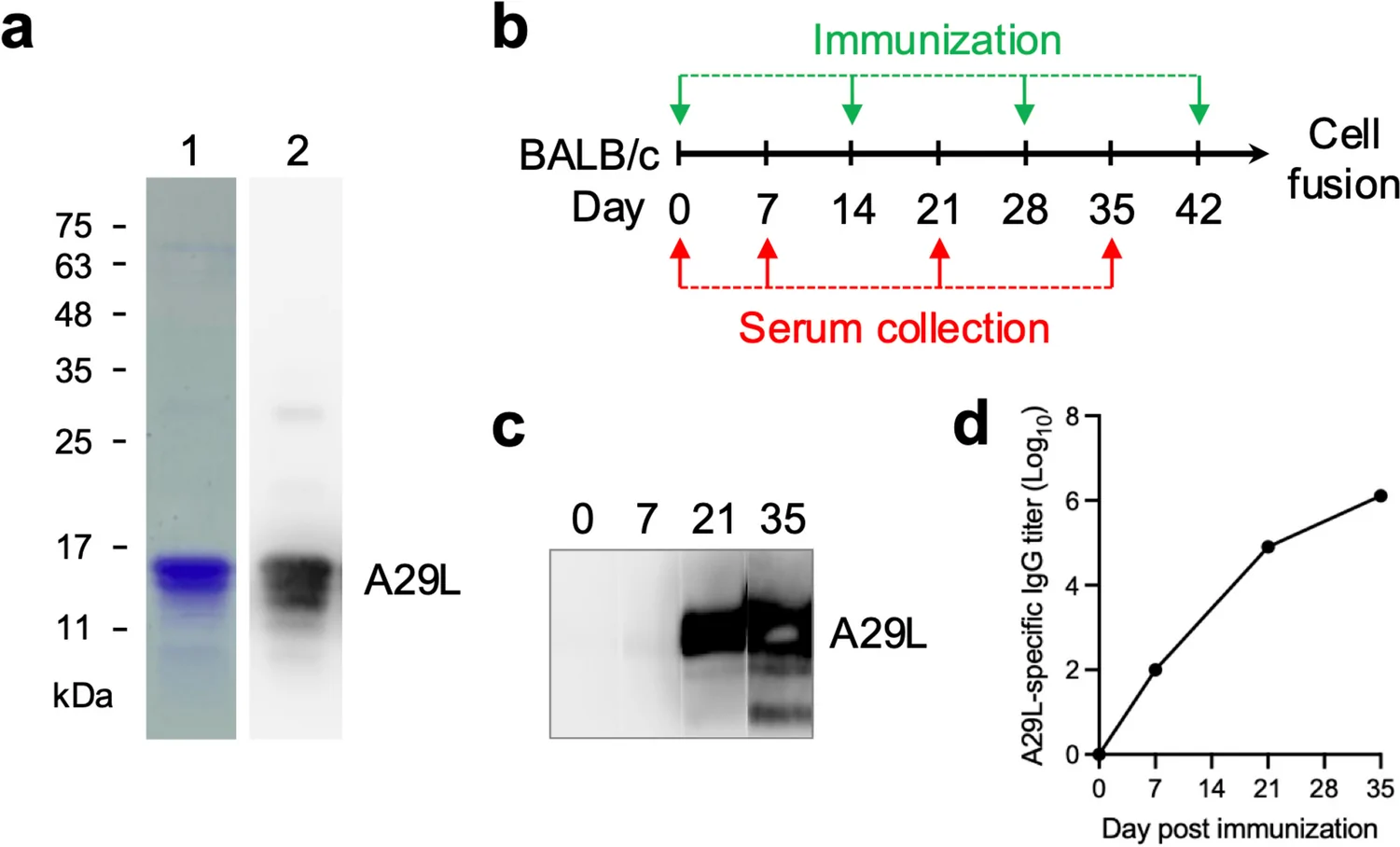
Immunization of mice with the recombinant MPXV A29L protein. **a** The pET28a plasmid containing the MPXV A29L cDNA was expressed in *E. coli* and purified for analysis of the purity by SDS-PAGE, coomassie staining (lane 1), and WB (lane 2). **b** The BALB/c mice were immunized with the purified A29L in a 2-week interval and the sera were collected at the indicated days as shown in the schematic diagram. **c** The collected antisera were subjected to WB for monitoring the induction of the A29L-specific antibodies. **d** The collected antisera were subjected to ELISA as described in the “Materials and methods**” s**ection for determination of the A29L-specific IgG titer

For the production of recombinant MPXV A29L protein, the pET28a-A29L-His plasmid was transformed into *E. coli* BL21(DE3). The purified A29L was analyzed by SDS-PAGE and Western blotting (WB) with the anti-His tag antibody (Fig. 1a). After immunization of BALB/c mice with the purified A29L protein (Fig. 1b), the antisera were collected for subsequent analysis of the A29L-specific immune response by WB and ELISA. The results indicated that the A29L-specific antibodies were significantly increased in the antisera collected on day 21 and day 35 (Fig. 1c and d).

### Production of the A29L-specific mAbs

**Fig. 2:**
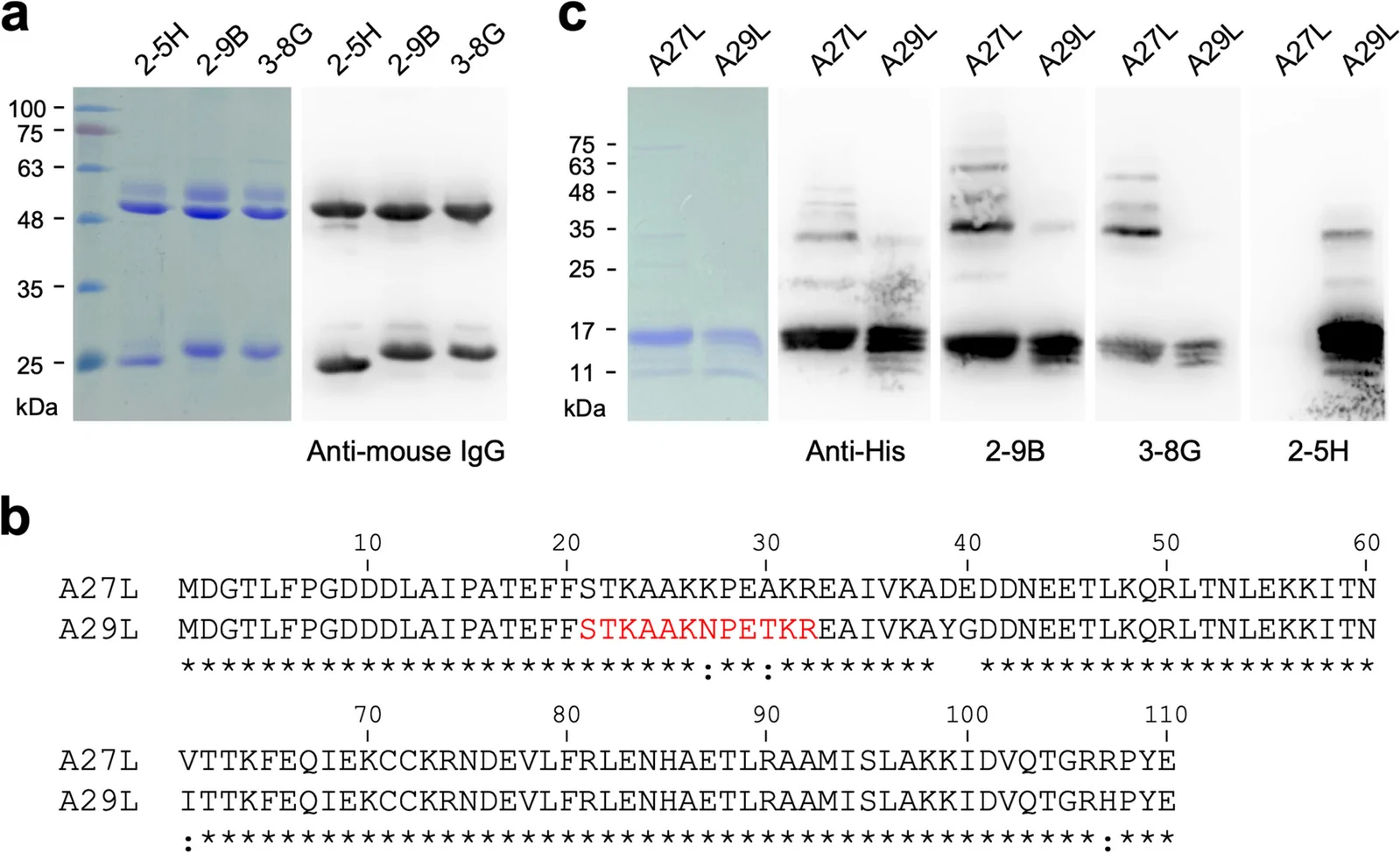
The specificity of the anti-MPXV A29L mAbs 2-9B, 3-8G, and 2-5H. **a** Three anti-MPXV A29L mAbs, including 2-9B, 3-8G, and 2-5H, were generated by the conventional hybridoma technology. The purity of mAbs 2-9B, 3-8G, and 2-5H was analyzed by SDS-PAGE, coomassie staining (left panel), and WB (right panel). **b** Alignment of the amino acid sequences of VACV A27L (Swiss-Prot: P11258.3) and MPXV A29L (GenBank: AAQ72865.1) by using the Clustal Omega program (Madeira et al. 2022). Asterisk (*) indicates positions which have a single, fully conserved residue. Colon (:) indicates conservation between groups of strongly similar properties. The residues of A29L HBD are marked in red color. **c** The purified A27L and A29L proteins were analyzed by SDS-PAGE, coomassie staining, and WB with anti-His tag antibody, 2-9B, 3-8G, or 2-5H, respectively

Following immunization with four doses of the purified A29L, splenocytes obtained from BALB/c mice were fused with Sp2/0-Ag14 cells to generate hybridoma cell lines. Subsequently, positive hybridoma clones were screened via ELISA, resulting in the selection and purification of three mAbs: 2-5H, 2-9B, and 3-8G (Fig. 2a).

### 2-5H is highly specific for MPXV A29L

MPXV A29L, sharing 94.5% sequence identity with VACV A27L (Fig. 2b), was utilized along with the recombinant A27L proteins to access the specificity of 2-5H, 2-9B, and 3-8G. The results of WB demonstrated that both 2-9B and 3-8G could bind to both A29L and A27L proteins (Fig. 2c). Notably, 2-5H exhibited higher specificity for A29L without displaying cross-reactivity with A27L (Fig. 2c). The *K*_D_ values of 2-9B, 3-8G, and 2-5H against MPXV A29L protein were 25.5 nM, 38.9 nM, and 12.5 nM, respectively, as measured by the ELISA method described in the “Materials and methods” section.

### The binding epitopes of 2-9B, 3-8G, and 2-5H

**Fig. 3:**
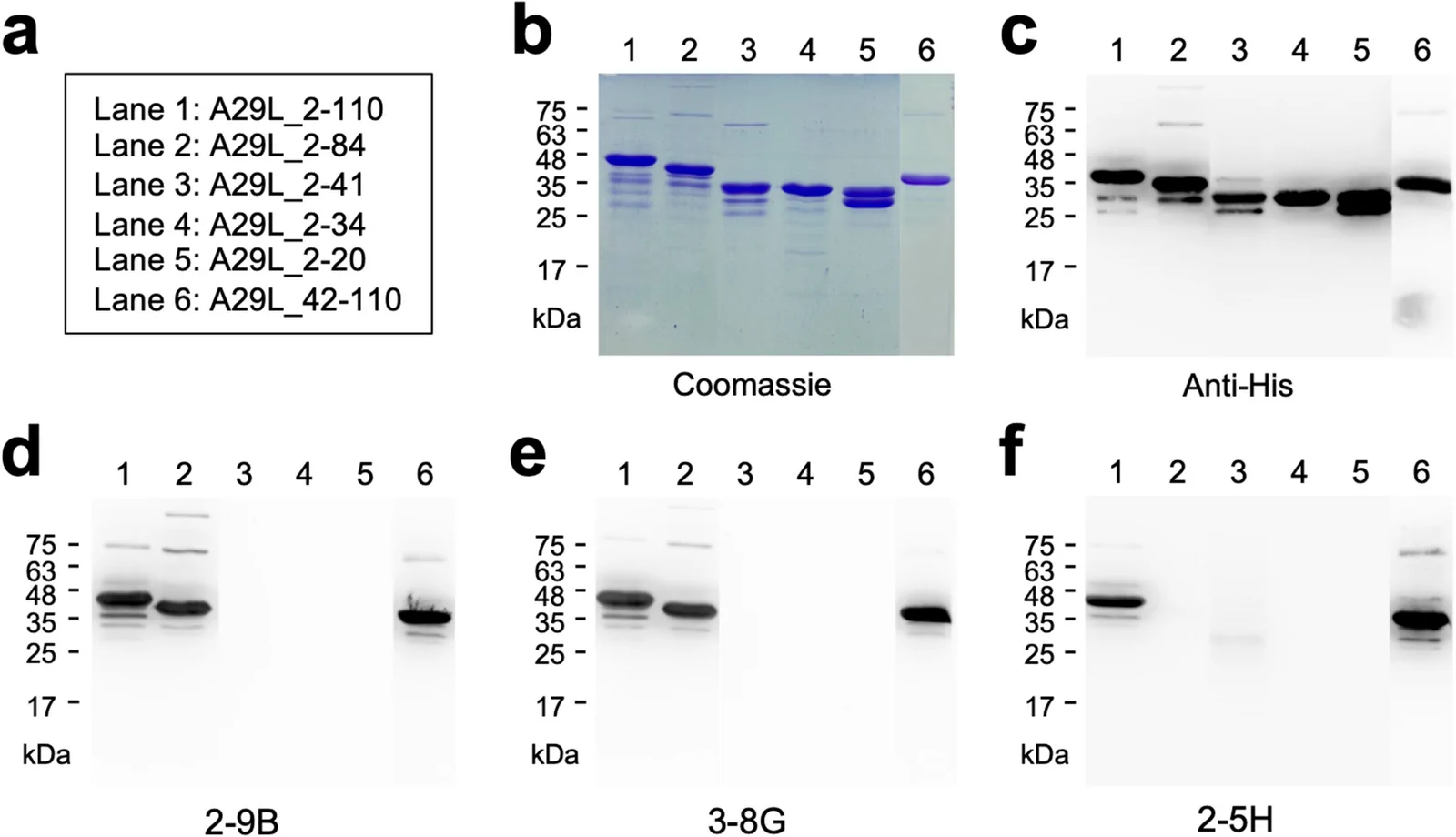
Characterization of the binding epitopes of 2-9B, 3-8G, and 2-5H. The truncated A29L fragments (lanes 1–6) were expressed as the His-tagged sfGFP fusion proteins (**a**), and further utilized for SDS-PAGE, coomassie staining (**b**), and WB with anti-His tag antibody (**c)**, 2-9B (**d**), 3-8G (**e**), or 2-5H (**f**), respectively

To characterize the binding epitopes of 2-9B, 3-8G, and 2-5H, a series of truncated A29L fragments (Fig. 3a) were employed for SDS-PAGE and coomassie staining (Fig. 3b), followed by WB using the indicated antibodies: anti-His tag antibody, 2-9B, 3-8G, and 2-5H, respectively (Fig. 3c–f). The results indicated that 2-9B and 3-8G can recognize A29L_2-110, A29L_2-84, and A29L_42-110 (Fig. 3d and e, lanes 1, 2, and 6), whereas no signal was observed in lanes 3–5, suggesting that the binding epitopes of 2-9B and 3-8G are located within residues 42–84 of A29L. Furthermore, 2-5H only binds to A29L_2-110 and A29L_42-110 (Fig. 3f, lanes 1 and 6), but not A29L_2-84 (Fig. 3f, lane 2), indicating that the binding epitopes of 2-5H are located within residues 85–110 of A29L. However, the sequence homology of residues 85–110 between A29L and A27L is highly conserved (Fig. 2b), except for the substitution of Arg-107 with His-107 in A29L. The difference at residue 107 may not be the key antigenic determinant responsible for 2-5H losing its capability of binding to A27L. Interestingly, a minor cross-reactivity of 2-5H with A29L_2-41 was observed (Fig. 3f, lane 3), indicating that a partial binding epitope of 2-5H is located in this region which might be sterically hindered by the 42–84 residues. It is noteworthy that residues 27, 30, 39, and 40 among A29L and A27L are not well conserved (Fig. 2b). Therefore, the specificity of 2-5H in distinguishing between A29L and A27L might be determined by its recognition of these non-conserved residues.

### 2-9B, 3-8G, and 2-5H cannot prevent A29L from binding to heparin

**Fig. 4:**
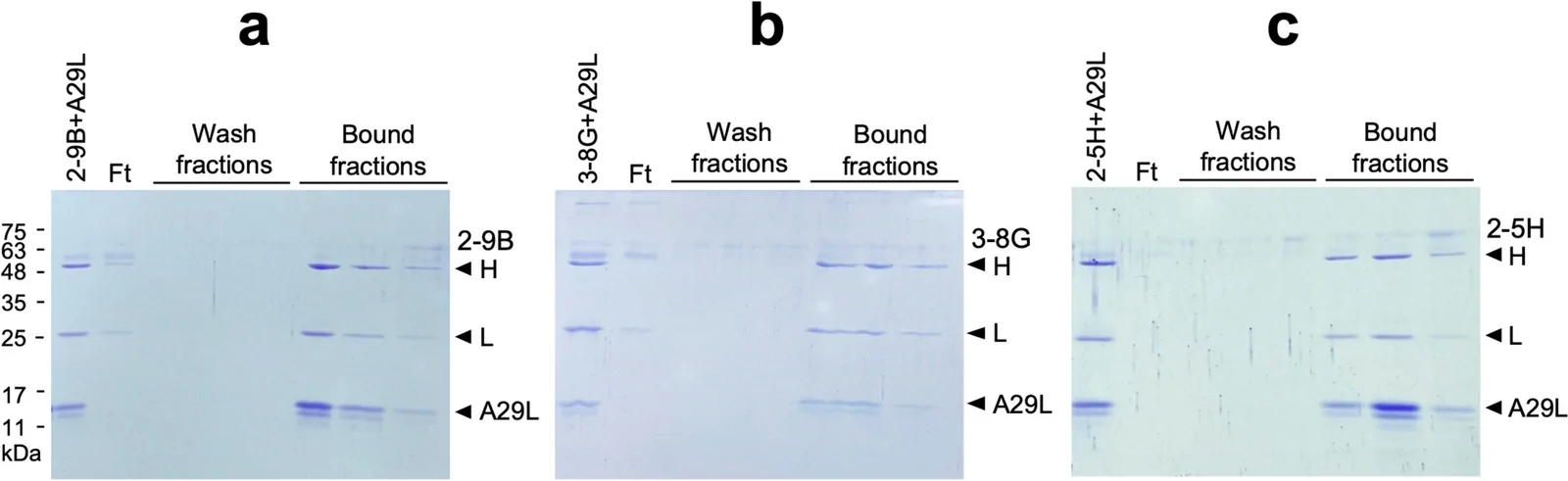
2-9B, 3-8G, and 2-5H cannot inhibit A29L binding to heparin column. The purified A29L protein was pre-incubated with 2-9B (**a**), 3-8G (**b**), or 2-5H (**c**), and then loaded on the HiTrap heparin HP column (Cytiva). The mAb-A29L mixtures, flowthrough fractions (Ft), wash fractions, and bound fractions were analyzed by SDS-PAGE and coomassie staining. H, antibody heavy chain. L, antibody light chain

It was shown that A27L facilitates viral attachment to host cells through its HBD in the N-terminal region (residues 21–32) (Chung et al. 1998; Hsiao et al. 1998). A29L, on the other hand, displays a robust affinity with glycosaminoglycans (GAGs), including heparin and chondroitin sulfate/dermatan sulfate (Shi et al. 2022). To investigate whether 2-5H, 2-9B, and 3-8G could interfere with A29L binding to heparin, these mAbs were incubated with A29L and subsequently loaded onto the heparin column. The fractions bound to the column were then eluted and subjected to SDS-PAGE, followed by coomassie staining. The results revealed that, when combined with A29L, 2-9B, 3-8G, or 2-5H were also retained on the heparin column and co-purified in the bound fractions (Fig. 4a–c). These data suggested that these three mAbs do not inhibit A29L binding to the heparin column and do not interfere with the function of HBD.

### 2-9B, 3-8G, and 2-5H did not exhibit neutralizing activity against MPXV

**Fig. 5:**
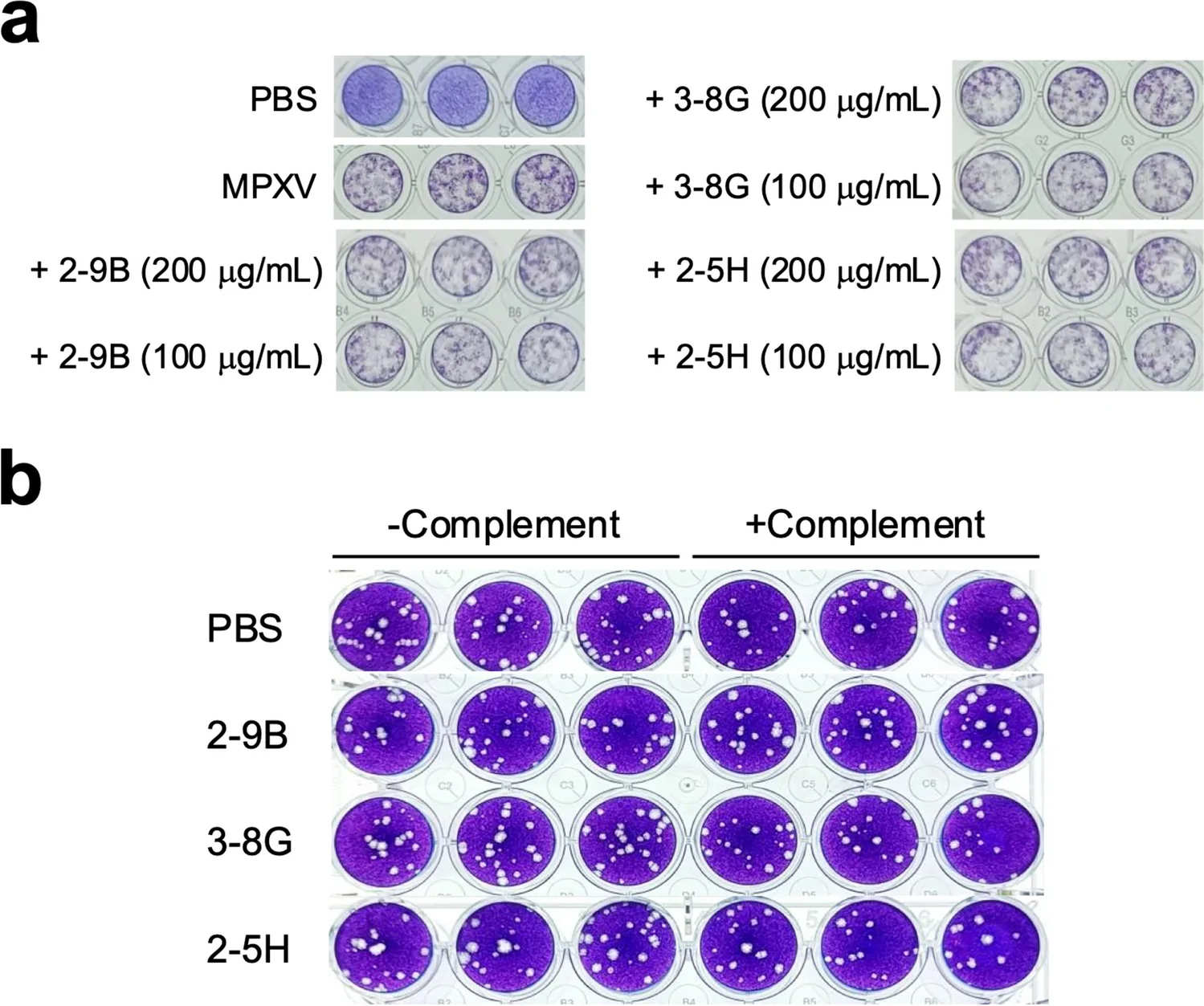
2-9B, 3-8G, and 2-5H cannot neutralize MPXV. **a** The MPXV virions were pre-incubated with PBS, 2-9B, 3-8G, or 2-5H (200 µg/mL or 100 µg/mL) and then added to Vero E6 cell monolayers for performing the NT assays. After incubation for 7 days at 37°C in the 5% CO_2_ incubator, the cells were fixed with formaldehyde and stained with crystal violet. Three independent experiments were conducted for each antibody at the designated concentration. **b** The MPXV virions were pre-incubated with PBS, 2-9B, 3-8G, or 2-5H (100 µg/mL) in the absence or presence of the 10% baby rabbit complements (± complement) and then added to Vero E6 cell monolayers for performing the PRNT assays. Three independent experiments were conducted for each antibody group

To investigate the neutralizing potential of 2-9B, 3-8G, and 2-5H against MPXV, these three mAbs were pre-incubated with MPXV before being added to the Vero E6 monolayers for the NT assays. As shown previously that 2-9B, 3-8G, and 2-5H were unable to inhibit A29L binding to heparin column, these three mAbs did not exhibit neutralizing activity against MPXV, as evidenced by the formation of plaques even in the presence of 2-9B, 3-8G, and 2-5H at concentrations of 100 or 200 µg/mL in the NT assays (Fig. 5a). It has been suggested that complement activation may play an important role in enhancing the neutralizing activity of both anti-VACV and anti-MPXV antibodies (Kaever et al. 2016). Thus, we further examined whether baby rabbit complements can enhance the neutralizing activity of 2-9B, 3-8G, and 2-5H. The results of plaque reduction neutralization test (PRNT) assays showed that these three mAbs did not exhibit better neutralizing activity for blocking MPXV infection in the presence of 10% baby rabbit complements (Fig. 5b).

### Specific detection of MPXV A29L by LFIA

**Fig. 6:**
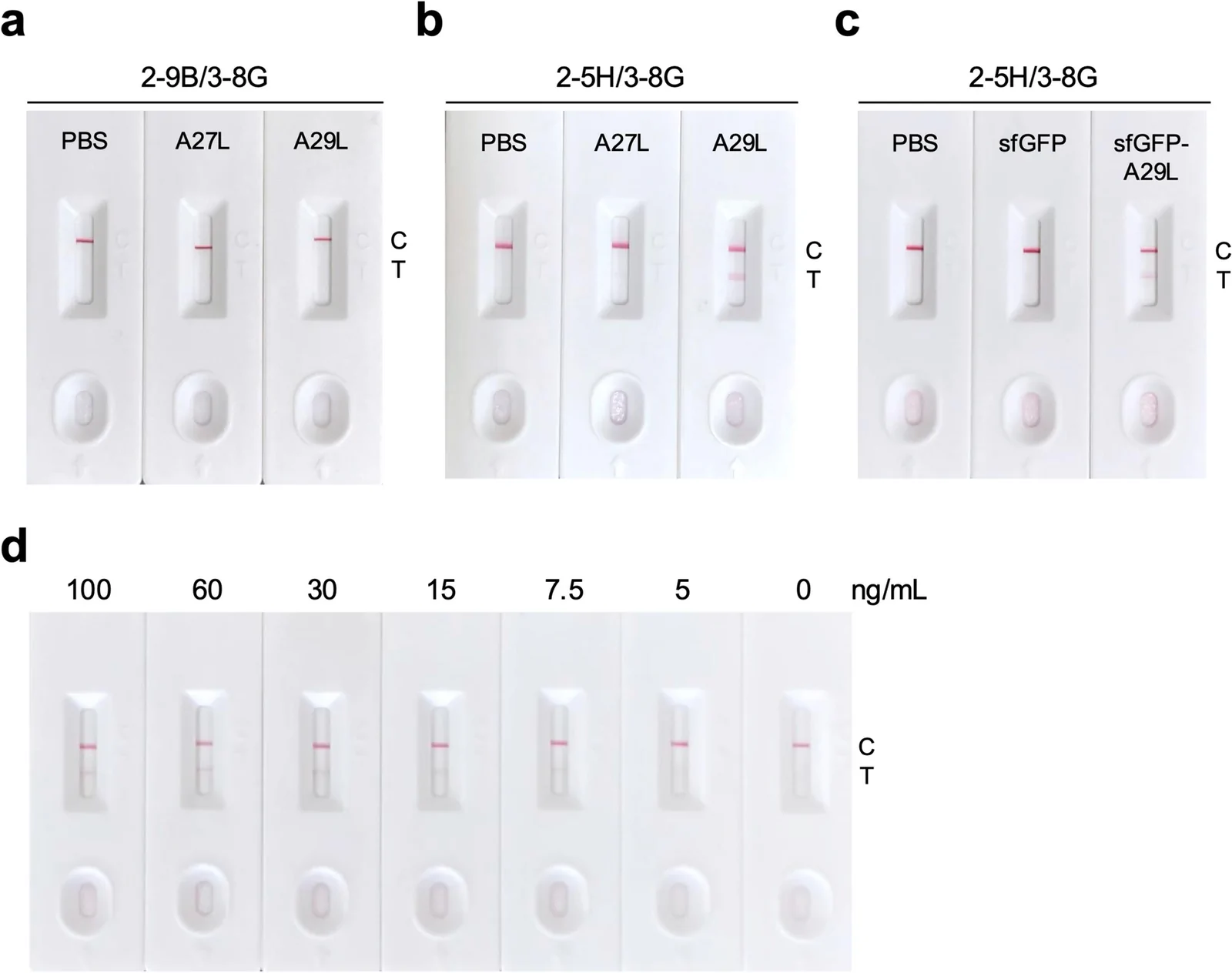
The LFIA rapid test for specific detection of MPXV A29L. The LFIA rapid test was composed of the 2-9B-conjugated (**a**) or 2-5H-conjugated (**b**) colloidal gold conjugate pad, 3-8G-coated test (T) line, and the protein G-coated control (C) line on the NC membrane. PBS, A27L protein (500 ng), or A29L protein (500 ng) was added to the sample pad for starting the LFIA rapid test, respectively. **c** The sfGFP or sfGFP-A29L (500 ng) was also used to examine the functional specificity of the 2-5H/3-8G LFIA rapid test. The valid C lines all clearly appeared in these rapid tests, whereas the 2-5H-conjugated colloidal gold captured in the T line was only seen while adding A29L or sfGFP-A29L to the sample pad. **d** The various amounts of A29L protein (100, 60, 30, 15, 7.5, 5, and 0 ng/mL) were added to the sample pads of the 2-5H/3-8G LFIA rapid tests for determining the values of the limit of detection (LOD)

For the development of the LFIA rapid test, the 2-9B-conjugated (Fig. 6a) or the 2-5H-conjugated (Fig. 6b) colloidal gold was coated on the conjugate pad, while the capture antibody 3-8G was coated on the test line of the NC membrane. In LFIA results, no detection signal was observed when using the 2-9B/3-8G antibody pairing for detection of PBS, A27L, and A29L (Fig. 6a). In contrast, a clear detection signal appeared on the test line when A29L was added to the LFIA rapid test which contained the 2-5H/3-8G antibody pairing (Fig. 6b). In addition, the sfGFP or sfGFP-A29L was also utilized to examine the specificity of the 2-5H/3-8G LFIA rapid test. The result showed that only sfGFP-A29L can be captured in the test line of the 2-5H/3-8G LFIA rapid test, indicating that this unique antibody pairing can be effectively utilized for the specific detection of MPXV A29L. The different amounts of A29L were further utilized in the LFIA rapid test for determining the values of the limit of detection (LOD). The results showed that the minimum concentration that can be detected by the 2-5H/3-8G LFIA rapid test is around 7.5–15 ng (Fig. 6d).

### The antibody profiles of Mpox patient sera against different A29L domains

**Fig. 7:**
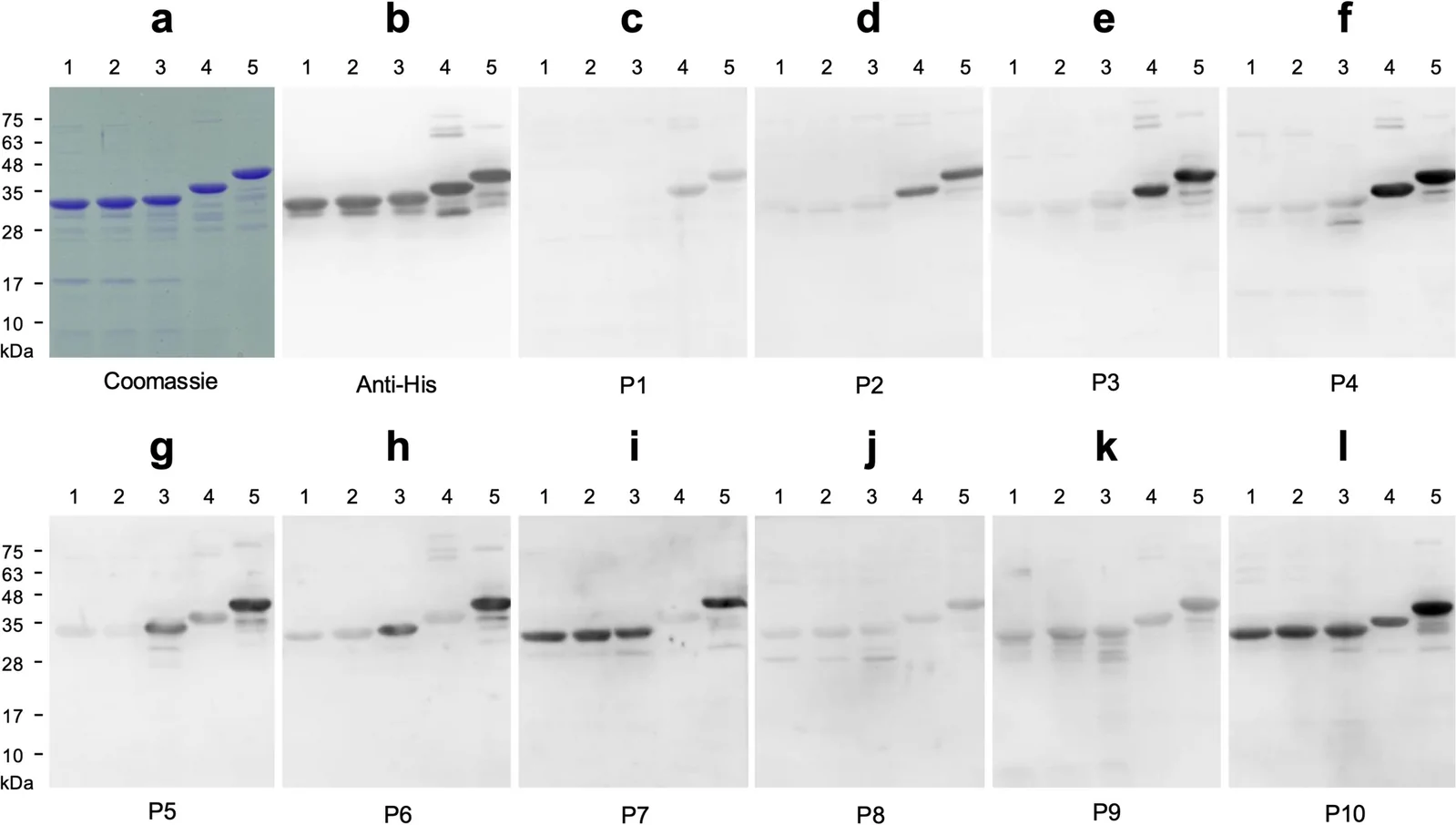
The antibody profiles of the Mpox patient sera against the truncated A29L fragments. The truncated A29L fragments were expressed as the His-tagged sfGFP fusion proteins, and further utilized for SDS-PAGE, coomassie staining (**a**), and WB with anti-His tag antibody (**b**), or the human sera (1:500) collected from Mpox patients P1–P10 (**c**–**l**), respectively. Lane 1, sfGFP fused with the 2–20 residues of A29L (30.6 kDa). Lane 2, sfGFP fused with the 2–34 residues of A29L (32.1 kDa). Lane 3, sfGFP fused with the 2–41 residues of A29L (32.9 kDa). Lane 4, sfGFP fused with the 42–110 residues of A29L (36.7 kDa). Lane 5, sfGFP fused with the full-length A29L (41.0 kDa)

It remains uncertain whether the human immune system can generate antibodies like 2-5H, 2-9B, and 3-8G. To address this, human sera collected from ten Mpox patients who have not received the smallpox-based vaccine were used to perform the WB against a series of truncated A29L fragments (Fig. 7). The results showed that the human sera from patients 1–4 (P1–P4) contained antibodies which were only capable of binding to the truncated peptide 42–110 and the full-length A29L, but barely recognized peptides 2–20, 2–34, and 2–41 (Fig. 7c–f). The human sera from P5 and P6 exhibited stronger binding to peptide 2–41 and the full-length A29L, without displaying clear cross-reaction with peptides 2–20, 2–34, and 42–110 (Fig. 7g and h). The human serum from P7 can only recognize peptides 2–20, 2–34, and 2–41 and the full-length A29L, without displaying clear cross-reaction with peptide 42–110 (Fig. 7i). Interestingly, the results of WB using P7 were opposite to the results of WB using P1–P4. The human sera from P8–P10 can recognize all of the truncated peptides and the full-length A29L, but the detection signal of WB using P8 was much lower than the results of WB using P9 and P10 (Fig. 7j–l). These findings suggest that the antibody profiles of Mpox patient sera against various A29L domains may vary significantly.

## Discussion

In this study, we generated the recombinant MPXV A29L protein (Fig. 1) which demonstrated heparin-binding capability (Fig. 4). This purified A29L protein was utilized to immunize BALB/c mice for generation of three anti-A29L mAbs, 2-9B, 3-8G, and 2-5H, by using the conventional hybridoma technology (Fig. 2a). Among these antibodies, 2-5H exhibited high specificity for MPXV A29L without displaying cross-reactivity with VACV A27L (Fig. 2c). Additionally, 2-5H demonstrated reduced but consistent binding to the peptide spanning from residues 2 to 41, along with a distinct binding epitope located in the region spanning from residues 42 to 110 of A29L (Fig. 3f). Previous findings have shown that a murine anti-VACV A27L mAb, 12C3, also recognized two distinct linear epitopes located in residues 21–40 and residues 81–100 (Kaever et al. 2016). These findings suggest that both MPXV A29L and VACV A27L possess a unique conformational epitope consisting of one N-terminal linear epitope and one C-terminal linear epitope. Moreover, due to the non-conservation of residues 27, 30, 39, and 40 between A29L and A27L, antibodies like 2-5H and 12C3 can serve to differentiate between these homologous proteins within the members of the *Orthopoxvirus* genus. In light of this, an antibody pairing comprising 2-5H and 3-8G was developed for the LFIA rapid test in the study. Experimental results demonstrated that the rapid test can exclusively detect samples containing the A29L protein (Fig. 6b), suggesting its potential application in diagnosing MPXV infection. Furthermore, it is observed that the LFIA rapid test comprising 2-9B and 3-8B cannot detect A27L and A29L (Fig. 6a). It is also noted that 2-9B and 3-8G bind to the same region of A29L (Fig. 3d and e). It is expected that 2-9B binding to A27L or A29L may interrupt the additional binding of 3-8G due to the steric hindrance. Therefore, there was no detection signal in the LFIA rapid test based on the 2-9B/3-8G antibody pairing. In the previous study (Li et al. 2022c), three mouse mAbs, 3A1, 8F8, and 2D, against the MPXV A29L were developed by immunization of 6–8-week-old BALB/c mice with purified A29L using the murine hybridoma technique. 3A1 and 9F8 showed decent binding activity to MPXV A29L, camelpox virus A27L, and the corresponding membrane proteins of taterapox virus. In contrast, 2D1 showed good binding activity only to MPXV A29L, and weak binding activity against camelpox and taterapox antigen proteins. Using 9F8 as the capture antibody and 3A1 as the detection antibody, the sandwich ELISA can detect all three orthopoxvirus antigens and the authentic VACV. In contrast, by using 3A1 as the capture antibody and 2D1 as the detection antibody in the ELISA, MPXV A29L can be specifically detected with the minimum detection limit of 128 pg/mL, but VACV could not be detected. Finally, the LFIA rapid test kit based on the 3A1-2D1 antibody pairing is still reactive to MPXV A29L antigen at a minimum concentration of 10 ng/mL. The mAb 2-5H developed in this work is also highly specific for MPXV A29L, without exhibiting cross-reactivity with VACV A27L (Fig. 6b). Therefore, we also found that the LFIA rapid test kit based on the 2-5H/3-8G antibody pairing displayed a great reactivity and specificity only to MPXV A29L at a minimum concentration of 7.5–15 ng/mL (Fig. 6c and d).

Previous studies have identified three major structural domains in both A27L and A29L, including HBD spanning residues 21–32, the coiled-coil domain (CCD) spanning residues 42–84, and the leucine zipper domain (LZD) spanning residues 84–110. It has been demonstrated that a specific group of VACV- or MPXV-neutralizing antibodies targets either the HBD or the regions adjacent to the HBD (Kaever et al. 2016; Li et al. 2023; Riccardo and Pablo 2023). Our findings indicate that 2-9B and 3-8G target the CCD of A29L, while 2-5H targets the LZD and an epitope that partially overlaps with the HBD of A29L (Fig. 3). As a result, it is not surprising that 2-9B, 3-8G, and 2-5H do not effectively inhibit A29L binding to heparin or block MPXV infection (Figs. 4 and 5). Interestingly, a study has reported that an anti-MPXV A29L mAb, 69–126-3–7, can prevent the interaction of A29L with heparin, but it does not have any effect on blocking the infectivity of MPXV (Hughes et al. 2014). Moreover, a set of anti-A27 HBD mAbs (1G6, 12G2, and 8H10) was demonstrated to neutralize IMV in an in vitro neutralization assay in a complement-dependent manner and provided protection against VACV challenge in mice (Kaever et al. 2016), suggesting that complement activation may play an important role in enhancing the neutralizing activity of both anti-VACV and anti-MPXV antibodies. In this work, we found that baby rabbit complements cannot enhance the virus-neutralizing activity of the anti-MPXV A29L mAbs, 2-9B, 3–8, and 2-5H, in the PRNT assays (Fig. 5b). In conclusion, despite recognizing very similar epitope peptide, the virus-neutralizing activity of HBD-specific antibodies can vary significantly and there is no correlation between neutralizing activity and the ability of blocking VACV interaction with chondroitin sulfate or heparan sulfate. It is likely that the virus can utilize other GAG-binding proteins to adhere to target cells (Matho et al. 2018). Therefore, multiple neutralizing antibodies targeting different surface antigens might be required for efficient inhibition of orthopoxvirus infection. Furthermore, studies have reported that the LZD of A27L is involved in interaction with an integral viral membrane protein, A17L, for anchoring in the viral membrane and packaging into IMV particles (Rodriguez et al. 1993; Vazquez et al. 1998; Wang et al. 2014). Further characterization is needed to determine whether 2-5H can disrupt the interaction between A29L and A17L.

It has been reported that an antigen capture ELISA utilizing anti-VACV A27L mAb A1/40 (as the capture antibody) and mAb A3/710 (as the detection antibody) can specifically detect orthopoxvirus isolates, including MPXV (Stern et al. 2016). However, this platform was unable to differentiate MPXV from other orthopoxviruses. In a separate study, a synthetic MPXV A29L_17–49_ polypeptide was conjugated to KLH protein and subsequently used to immunize mice to obtain splenocytes for generating hybridoma cells (Ye et al. 2023). A mAb pairing comprising 7C5 and 5D8 with specificity targeting the N-terminal residues 17–49 of MPXV A29L was employed in an LFIA test strip that exhibited no cross-reactivity with VACV A27L or cowpox virus 162 protein (Ye et al. 2023). This study illustrated that mAbs targeting the N-terminal residues 17–49 of A29L can be effectively utilized in the MPXV-specific LFIA rapid test kit. Additionally, the previously mentioned mAb 69–126-3–7 (α-A29), which binds specifically to MPXV A29L without displaying cross-reactivity with the homologous proteins from other orthopoxviruses, was acquired and employed as the primary antibody to develop an immunodiagnostic assay for specifically detecting MPXV A29L (Davis et al. 2023). Furthermore, our finding demonstrated that the mAb 2-5H, recognizing an epitope spanning residues 21 to 41 of A29L and exhibiting no cross-reactivity with VACV A27L, is a valuable tool for application in the MPXV-specific LFIA rapid test (Fig. 6). To enhance the sensitivity of MPXV detection, a multilayered SiO_2_-Au core dual-quantum dot (named SiO_2_-Au/DQD) was developed for fluorescence-enhanced dual-signal LFIA. This innovative approach operates with a portable fluorescence reader, enabling qualitative or quantitative analysis of MPXV A29L (Yang et al. 2023). The Si-Au/DQD-based LFIA was reported with detection limits of 0.5 and 0.0021 ng/mL for the colorimetric and fluorometric modes, and was 238- and 3.3-fold more sensitive than the conventional AuNP-based LFIA and ELISA methods, respectively (Yang et al. 2023). Taken together, early diagnosis plays a crucial role in preventing MPXV epidemics and minimizing close contact with infected humans and animals. Combining PCR-based platforms with the ELISA-based or LFIA-based methods, along with clinical symptoms, can offer more accurate diagnosis of MPXV infection.

A series of truncated A29L fragments were generated for epitope mapping in the study. These truncated A29L fragments were also utilized to analyze the antibody profiles of sera collected from Mpox patients. The data showed that Mpox patient sera did not contain high levels of antibodies binding to the target peptide spanning residues 2 to 20 of A29L (Fig. 7c–g). It has been reported that the N-terminal 20 residues of the purified A27L on mature virion were possibly removed (Takahashi et al. 1994). Our serological data seem to corroborate this finding. Therefore, it is reasonable to infer that the antibodies targeting the N-terminal 20 residues of A29L may not be strongly induced following MPXV infection in humans. Although our analysis was based on ten Mpox cases, the results still demonstrated that sera collected from Mpox patients who had not received the smallpox-based vaccine contained significantly lower levels of antibodies targeting the N-terminal 2–34 residues of A29L. This suggests that the C-terminal portion of A29L is likely more immunogenic (Fig. 7).

In conclusion, our study demonstrated that A29L-specific IgGs can indeed be induced upon MPXV infection in humans. However, the antibody profiles observed in Mpox patient sera varied with the recognition of different structural domains of A29L. Consequently, A29L emerges as an ideal MPXV protein for serological assays, shedding light on the extent of MPXV infection and effectiveness of vaccines. It is also important to note that anti-A29L mAb may not necessarily block MPXV infection solely based on their functionality in preventing A29L binding to the host cell receptors. It is possible that they need to be combined with antibodies targeting other MPXV proteins to achieve stronger viral neutralizing potency. The limitation of the study also includes that the A29L antigen utilized for immunization of mice in this work was prepared by the *E. coli* expression system. This recombinant A29L protein, which lacks glycosylation, may not exist as a correct conformation like the authentic A29L on the MPXV surface. The produced mAbs by hybridoma techniques may fail to recognize MPXV virions or exhibit lower neutralizing potency. In addition, the unglycosylated A29L may influence the binding to heparin or the recognition by the clinically relevant patient antibodies. Furthermore, the binding epitopes of 2-9B, 3–8, and 2-5H only cover two limited regions of the A29L protein. The diversity of the mAbs should be increased for getting more potent neutralizing antibodies and identifying potential neutralizing epitopes. Even though the scope of the study can be further improved, based on our experimental results, anti-A29L mAbs were proven to be reliable tools for the specific detection of MPXV and for distinguishing orthopoxvirus infection.

## Data Availability

All data associated with this study are included in the paper.
